# The prognostic role of staging [18F]PSMA-1007 PET/CT volumetric and dissemination features in prostate cancer

**DOI:** 10.1007/s12149-025-02026-7

**Published:** 2025-02-17

**Authors:** Domenico Albano, Alessandro Temponi, Francesco Bertagna, Nazareno Suardi, Anna Talin, Marco Lorenzo Bonù, Luca Triggiani

**Affiliations:** 1 Nuclear Medicine, University of Brescia, Brescia, Italy; 2 Nuclear Medicine Department, ASST Spedali Civili di Brescia, Brescia, Italy; 3 Clinical Engineering, ASST Spedali Civili of Brescia, Brescia, Italy; 4 Department of Urology, University of Brescia, Brescia, Italy; 5 Radiation Oncology, ASST Spedali Civili di Brescia, Brescia, Italy

**Keywords:** Prostate cancer, PET/CT, Dmax, PSMA, Nuclear medicine

## Abstract

**Background:**

This study aimed the role of volumetric and dissemination features of staging [18F]PSMA-1007 PET/CT in predicting progression‐free survival (PFS) in patients with prostate cancer (PCa) and their relationship with the main clinical data (ISUP grade groups, number of lesions, PSA).

**Methods:**

We included 164 patients with high-risk PCa who underwent baseline [18F]PSMA-1007 PET/CT. With the help of LIFEx version 7.7, the main volumetric and dissemination PET parameters were semi-automatically extracted: PSMA-prostate tumor volume (PSMA-TV), PSMA-prostate total lesion (PSMA-TL), PSMA total TV (PSMA-TTV), PSMA total TL (PSMA-TTL) and Dmax corrected for body-surface-area (Dmax_bsa_). Spearman rank correlations between semiquantitative PET features and the clinical variables were analyzed. PFS estimates were plotted with the Kaplan–Meier method.

**Results:**

A high correlation was seen between the number of lesions and both PSMA-TTL (r 0.725), and Dmaxbsa (r 0.935). A moderate correlation was registered between PSA and PSMA-TTV (r 0.333), PSMA-TTL (r 0.441), Dmax_bsa_ (r 0.333), as well as between number of lesions and PSMA-TTV (r 0.342).

After a median follow-up of 17 months (range 2–45), relapse/progression happened in 17 patients (10%). PSA level, presence of distant metastases at staging, PSMA-TV, PSMA-TL, PSMA-TTL and Dmax_bsa_ were significantly associated with PFS at univariate analysis, but only the presence of distant metastases, PSMA-TTL and Dmax_bsa_ were confirmed to be independent prognostic factors.

**Conclusion:**

Volumetric and dissemination features derived by staging [18F]PSMA-1007 PET/CT were significantly correlated with PSA and number of lesions. The combination of PSMA-TTL and Dmax_bsa_ was the best predictor of PFS and may help to better stratify PCa patients.

## Introduction

Prostate-specific membrane antigen (PSMA) positron emission tomography/computed tomography (PET/CT) is a non-invasive diagnostic tool with superior accuracy over conventional imaging in studying prostate cancer (PCa) [1]. PSMA PET/CT is an examination exploiting the higher expression of PSMA by PCa cells compared to healthy prostatic tissue. Such exams have already been widely explored for staging purposes [2], both for recurrent [3] and newly diagnosed diseases. After a substantial body of evidence was accumulated regarding [68 Ga]Ga-PSMA imaging, recent advances in logistics and the enhanced availability of [18F]PSMA-1007 have significantly broadened their use in PSMA PET imaging for PCa. Indeed, [18F]PSMA-1007 shows the best diagnostic performances in the study of local disease, due to the prevalent non-urinary excretion, but incidental non-neoplastic bone uptakes reported in the literature, called unspecific bone uptake (UBU), are quite frequent and their meaning not yet clear [4, 5].

Beyond qualitative analysis [1], (semi)quantitative parameters derived from PET images are emerging as potentially useful in stratifying better PCa patients. Among them, standardized uptake value (SUV) is the parameter more commonly used due to its velocity and ease of extraction [6], but also more influenced by external factors, as confirmed by decades of studies with fluorodeoxyglucose (FDG) PET/CT. For these reasons, other quantitative features expressing tumor burden have been studied, like PSMA tumor volume (PSMA-TV) and PSMA total lesion (PSMA-TL). The prognostic role of these parameters was explored in several researches, especially in metastatic castration-resistant prostate cancer (mCRPC) receiving [177Lu]PSMA-617 therapy [7–9], second-line chemotherapy with cabazitaxel [10] and taxane-based chemotherapy (docetaxel) [11, 12]. In these analyses, high PSMA uptake seems to be associated with worse outcomes. So far, less data and heterogeneous are available for locally confined disease and primary staging of PCa [13, 14]. Recently, a new FDG PET-derived parameter was introduced for studying hematological malignancies and predicting their survival: the maximum tumor dissemination (Dmax) [15]. Dmax is a simple three-dimensional variable that represents the maximal distance between the two farthest PET lesions with increased uptake. Moreover, only preliminary and scarce findings are available about the potential role of Dmax in PCa [16, 17].

Therefore, this retrospective study aimed to investigate the usefulness of volumetric and dissemination [18F]PSMA-1007 PET/CT features measured at staging in predicting the outcome in PCa. Second aim was to investigate the correlation between different quantitative PSMA parameters, with the main clinical factors (such as Gleason grade, number of lesions, PSA values).

## Materials and methods

### Patients

This study was a monocentric retrospective study that analyzed all patients who underwent [18F]PSMA-1007 PET/CT between January 2021 and January 2024. Inclusion criteria were: (1) histologically confirmed PCa; (2) availability of staging [18F]PSMA-1007 PET/CT; (3) at least 12 months as follow–up; (4) not having received any treatment for prostate cancer before PET/CT (not androgen deprivation therapy, chemotherapy or radiotherapy). Instead, the exclusion criteria were: (1) a history of treatment for prostate cancer; (2) PSMA not avid primary prostatic tumor; (3) metastatic castration-resistant prostate cancer (mCRPC) patients. Finally, 164 patients were recruited in this study. For each patient, the main clinical and epidemiological data were collected, such as age, PSA value at diagnosis, ISUP grade group (1–3 considered as early, 4–5 as advanced), ECOG performance status, lactate dehydrogenase (LDH) level, alkaline phosphatase (ALP) level, international society of urological pathology (ISUP) grading and clinical stage. The study adhered to the Declaration of Helsinki and was approved by the local ethics committee. Written informed consent was provided by the recruited patients. The patients were treated according to the institutional protocols at the time of the diagnosis, following the main international guidelines.

### [18F]PSMA-1007 PET/CT imaging and interpretation

[18F]PSMA-1007 PET/CT scans were acquired about 90 min (range, 80–110 min) after the administration of a median dose of 305 MBq (279–340 MBq) of [18F]PSMA-1007, according to the current guidelines [1]. All PET/CT studies were performed using dedicated state-of-the-art tomographs Discovery ST and 690 (GE Healthcare). CT images acquisition parameters: 120 kV, 30–400 mA, and 0.984:1/39.37 pitch.

All PET/CT scans were revised for a visual and semiquantitative point of view by an experienced nuclear medicine physician with more than 10 years of experience (DA). who was blinded to the patient’s outcome. For visual analysis, every area of increased focal uptake higher than the background was reported as suspected of malignancy. Images were evaluated for the detection of the primary tumor, locoregional and extra-pelvic lymph nodes, bone and visceral metastases. For semiquantitative analysis, the feature extraction was performed using LIFEx version 7.7 [18]. By using the MTV protocol in this software, areas with PSMA uptake SUVmax ≥ 2.0 were segmented automatically in the whole body obtaining volumes of interest (VOI). Later, all VOIs were visually checked to confirm their pathological meaning or to remove in case of sites of physiologic (such as kidneys, bladder, intestine…) or benign uptakes according to PSMA-rads criteria [19]. To exclude UBU, a frequent unspecific finding with [18F]PSMA-1007, we applied the BUMP score as suggested in the literature [4]. The SUVmax threshold of 41% was applied to all remaining VOIs thought to be related to prostate cancer. Subsequently, we derived the PSMA-prostate tumor volume (PSMA-TV) as the volume measured in the primary prostatic lesion or lesions in case of multifocality of disease, PSMA-prostate total lesion (PSMA-TL) determined as PSMA-TV x SUVmean, the PSMA total tumor volume (PSMA-TTV) as the sum of all PSMA-TV values of the tumor-associated VOIs (prostatic lesion, metastatic lymph nodes and distant metastases), and PSMA total TL (PSMA-TTL) value was determined as the sum of all PSMA-TL values. Subsequently for the calculation of Dmax, LIFEx software applying the Euclidean formula measured the distance between all pairs of lesions (including all lesions) recording the greatest lesion distance. Subsequently, Dmax was normalized by the patient body surface area (_bsa_) calculated according to the Du Bois method to get Dmaxbsa. In the case of PET/CT with only prostate lesion, Dmax was defined as 0. For the calculation of Dmax, UBUs were excluded.

### Statistical analysis

MedCalc version 18 for Windows (Ostend, Belgium) was the software that we used for our statistical analyses. The categorical variables were described as simple and relative frequencies, while the numeric variables were described as average, median, standard deviations (SD) and range. To evaluate the correlation between volumetric/dissemination PET features and the main clinical-epidemiological variables (PSA, number of lesions, Gleason grade) we utilized Spearman’s rank correlation coefficient. A *p* value of 0.05 was considered statistically significant.

Receiver operating characteristic (ROC) curve was plotted to estimate the optimal thresholds values of PSMA-TV, PSMA-TL, PSMA-TTV, PSMA-TTL, DMAX_bsa_ for predicting PFS. PFS was estimated with the Kaplan–Meier method and log-rank test. PFS was defined as the interval from the date of diagnosis until recurrence or progression (considered as biochemical if PSA value increased again after treatment and/or structural defined as enlargement of original lesion or appearance of new lesions at imaging studies). Univariate and multivariate analyses were carried out using the Cox proportional hazard model to evaluate factors that predict PFS. Confidence interval (CI) was selected as 95% and a two-sided *p* value of less than 0.05 was accepted as significant.

To avoid multicollinearity impact between PET metrics, PSMA-TV and PSMA-TL were considered separately in the multivariate analysis. The same behavior for PSMA-TVV and PSMA-TTL.

## Results

### Population features and PET/CT findings

A total of 164 males (average age 70.4 years) were enrolled. The main patient’s characteristics are outlined in Table 1. The mean PSA level was 31.6 ng/ml ranging from 1.63 to 490 ng/ml, the mean ALP level was 74 U/L ranging from 39 to 102 U/L, and the mean LDH level was 186 U/L ranging from 125 to 225 U/L. Most patients had advanced ISUP grade (*n* = 108) and ECOG performance status was 0 in 80% of cases. [18F]PSMA-1007 PET/CT revealed no evidence of metastases in 111 patients (67%), whereas the remaining 53 (33%) patients had PSMA-avid metastases. Among them, 48 had skeletal metastases and 5 had abdominal lymph nodal metastases. UBUs were registered in 36 cases (22%) and in 27 patients were the only bone uptake reported in the scan.

**Table 1:** Baseline features of our population (164 patients)

	Patients *n* (%)	Average ± SD	Median (range)
Age		70.4 ± 7.8	72 (48–87)
ISUP grade			
1–3	56 (34%)		
4–5	108 (66%)		
ECOG performance status			
0	131 (80%)		
1–2	33 (20%)		
M status at staging			
M0	116 (81%)		
M1	48 (29%)		
D'amico risk classification			
Low-intermediate	19 (12%)		
High	145 (88%)		
PSA level (ng/mL)		31.6 ± 52.7	13(1.63–490)
ALP level (U/L)		74 ± 29.3	77.5 (39–102)
LDH level (U/L)		186 ± 43.6	185 (125–225)
PSMA-TV (cm^3^)		8.2 ± 7.8	5 (1.5–46)
PSMA-TTV (cm^3^)		87.9 ± 119	49.5 (3.5–880)
PSMA-TL		35.9 ± 75.7	9.95 (1.5–511)
PSMA-TTL		232.7 ± 404	90 (5–2560)
Dmax_bsa (_cm)		7.7 ± 10.2	4.6 (0–41.5)
Therapies after PET/CT			
Local			
Surgery	44 (27%)		
Radiotherapy	40 (24%)		
Radiotherapy + ADT	32 (20%)		
Systemic			
ADT	19 (12%)		
ADT + ARPI	20 (12%)		
ADT + Chemotherapy	9 (5%)		
Follow-up (months)		16 ± 9.5	14 (2–45)

*ADT* androgen deprivation therapy, *ARPI* androgen receptor signaling inhibitor, *LDH* lactate dehydrogenase, *ALP* alkaline phosphatase, *ISUP* international society of urological pathology, *PSA* prostate specific antigen, *ECOG* Eastern cooperative oncology group, *PSMA-TV* PSMA-prostate tumor volume, *TL* prostate total lesion, *TTV* total tumor volume, *TTL* total TL

After PET/CT, patients received local or systemic treatment according to the clinical features and stage: 44 underwent prostatectomy, 72 radiotherapy (with ADT in 32 cases, without ADT in 40), 19 only ADT, 20 ADT + androgen receptor signaling inhibitor and 9 to ADT plus chemotherapy. The median PSMA-TV was 5 cm^3^ (range 1.5–46 cm^3^), median PSMA-TL 9.95 (1.5–511), median PSMA-TTV 49.5 cm^3^ (3.5–880 cm^3^), median PSMA-TTL 90 (5–2560) and median Dmax_bsa_ 4.6 (0–41.5).

### Correlation between PET-quantitative parameters and clinical features

**Fig. 1:**
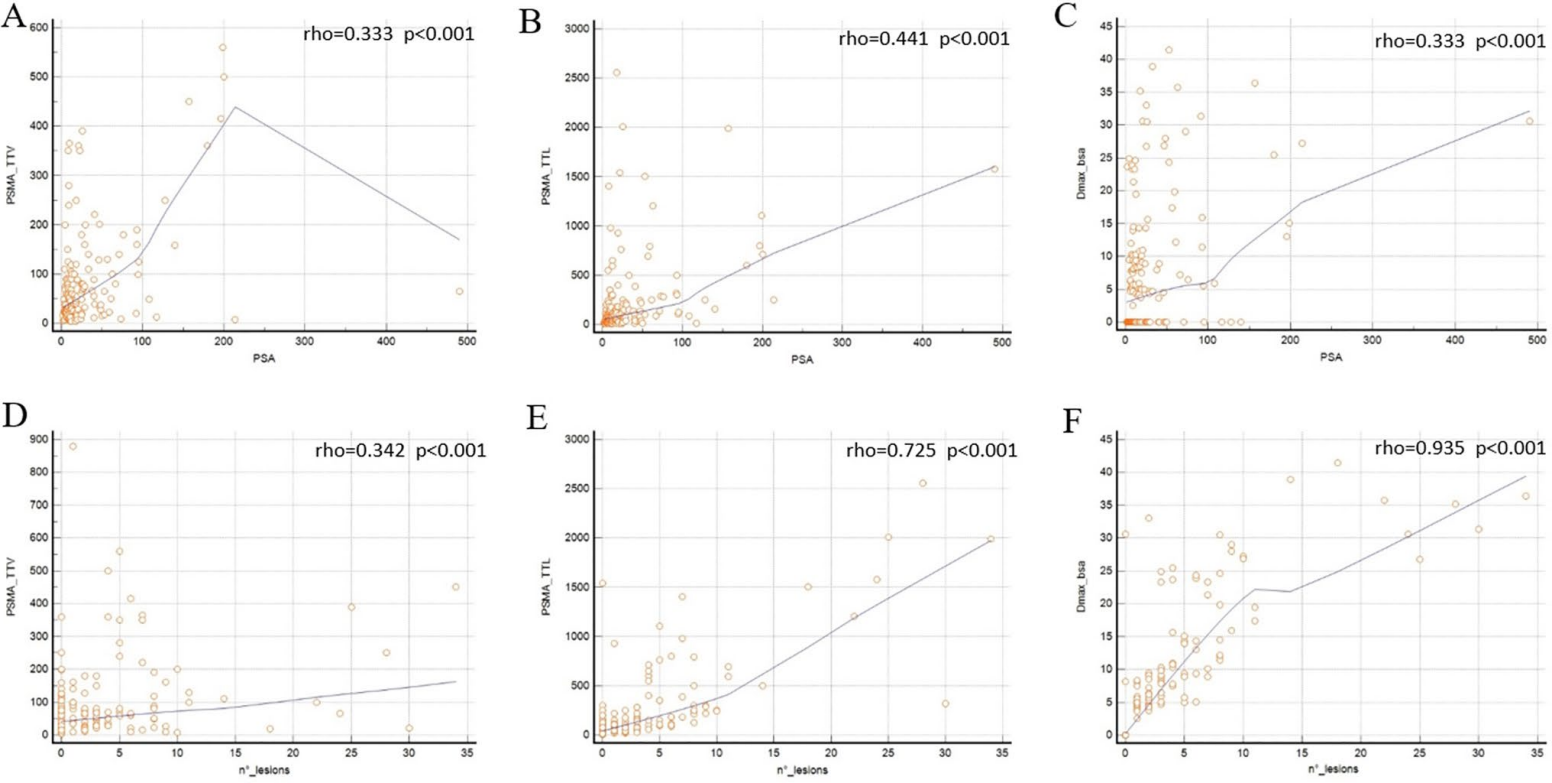
Correlation between PSA and PSMA-TTV (**A**), PSMA-TTL (**B**) and Dmax (**C**). Correlation between number of lesions (n°) and PSMA-TTV (**D**), PSMA-TTL (**E**) and Dmax (**F**)

Among the quantitative PSMA PET parameters investigated, several (PSMA-TTV, PSMA-TTL and Dmaxbsa) were significantly associated with PSA value and number of lesions (Fig. 1) (all *p* value < 0.001). A strong correlation was present among number of lesions and PSMA-TTL (Spearman rho 0.725), and among the number of lesions and Dmax_bsa_ (Spearman rho 0.935); instead a moderate correlation was registered between PSA and PSMA-TTV (Spearman rho 0.333), PSA and PSMA-TTL (Spearman rho 0.441), PSA and Dmax_bsa_ (Spearman rho 0.333), number of lesions and PSMA-TTV (Spearman rho 0.342). Instead other PET features, such as PSMA-TV and PSMA-TL, were not associated with the clinical variables evaluated (*p* > 0.05). The same for the association between all PET parameters and GG (*p* > 0.05).

### Prognostic role of [18F]PSMA-1007 PET/CT in predicting progression-free survival

**Fig. 2:**
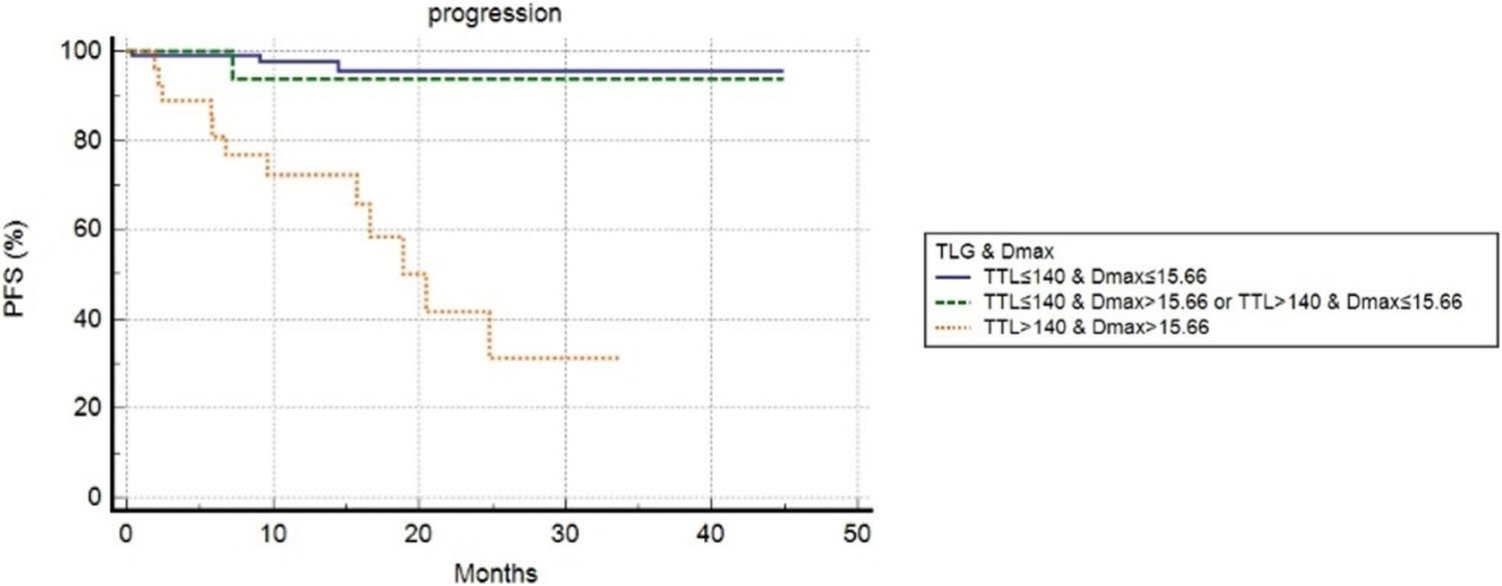
Progression-free survival curves according to PSMA-TV (**A**), PSMA-TL (**B**), PSMA-TTV (**C**), PSMA-TTL (**D**), Dmax_bsa_ (**E**)

**Fig. 3:**
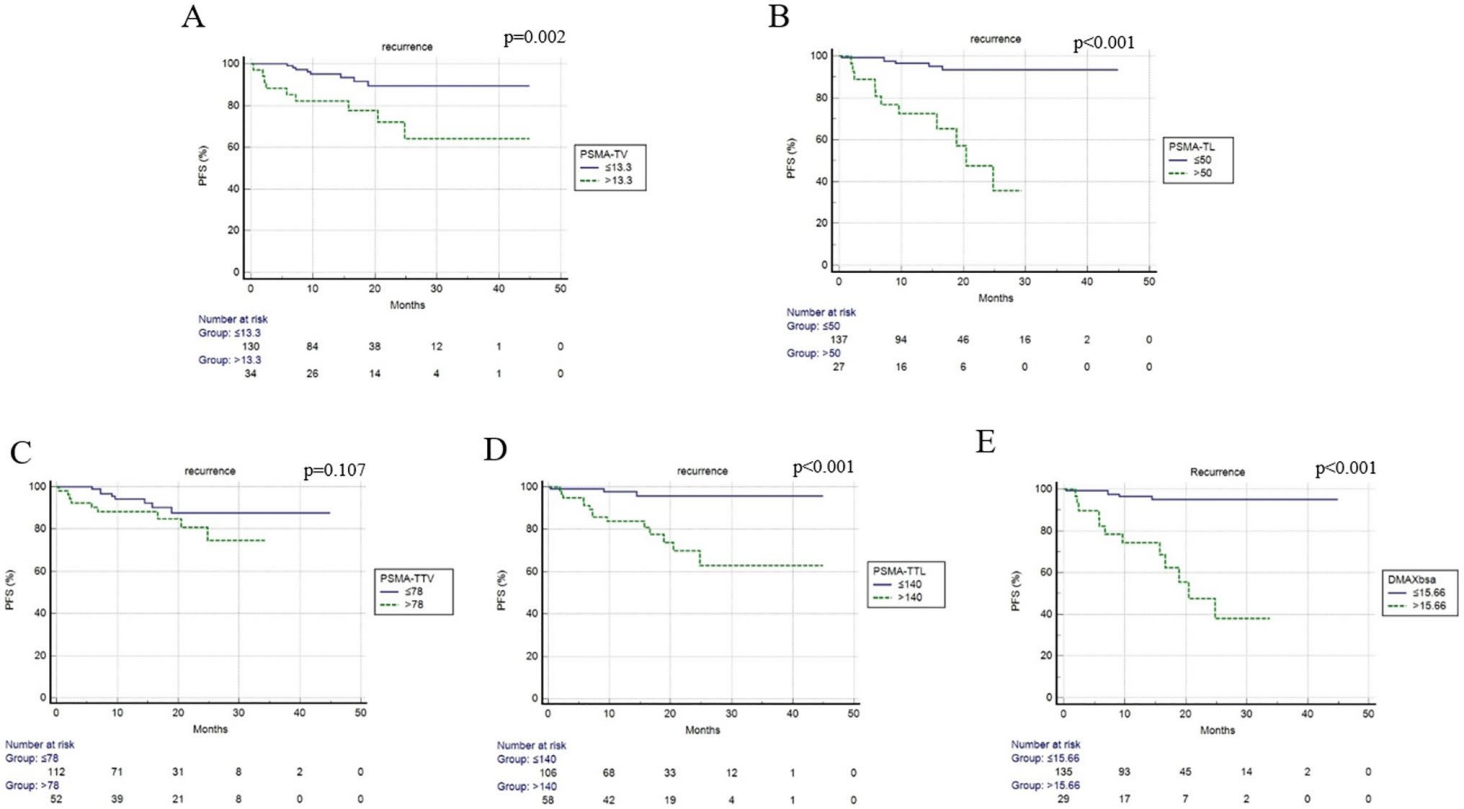
Progression-free survival curves according to the combination of PSMA-TTL and Dmax_bsa_. Blue line represents patients with low PSMA-TTL and low Dmax_bsa_; green line patients with low PSMA-TTL and high Dmax_bsa_ or high PSMA-TTL and low Dmax_bsa_; yellow line patients with high PSMA-TTL and high Dmax_bsa_

For the prognostic evaluation of the role of volumetric and dissemination features, we dichotomized these variables applying ROC curve analysis. The best threshold derived was represented in Table 2. At a median follow-up of 17 months (range 2–45), relapse or progression of disease occurred in 17 patients (10%) with a median time of 10 months (range: 2–25 months) from the staging [18F]PSMA-1007 PET/CT. One-year and 3-year PFS were 93% and 90%, respectively. At univariate analysis, PSA level, presence of distant metastases at staging, PSMA-TV, PSMA-TL, PSMA-TTL and Dmax_bsa_ were significantly correlated with the risk of progression/relapse (Table 3, Fig. 2). The other clinical features and PSMA-TTV were not associated with PFS. At the multivariate test, only distant metastases, PSMA-TTL and Dmax_bsa_ were confirmed to be independent prognostic factors concerning PFS. We combined PSMA-TTL and Dmax_bsa_ to derive three groups: (1) with low PSMA-TTL(≤ 140) and low Dmax_bsa_(≤ 15.66); (2) low PSMA-TTL(≤ 140) + high Dmax_bsa_ (> 15.66) or high PSMA-TTL(> 140) + low Dmax_bsa_ (≤ 15.66); (3) high PSMA-TTL(> 140) and low Dmax_bsa_ (> 15.66). This score stratified better patients PFS as shown in Fig. 3. Patients with high PSMA-TTL and high Dmax_bsa_ had lower PFS compared to the other two groups. The median PFS of patients with high PSMA-TTL and high Dmax_bsa_ was 10.3 months, of the patients with low PSMA-TTL + high Dmax_bsa_ or high PSMA-TTL + low Dmax_bsa_ was 15.2 months, of patients with low PSMA-TTL and low Dmax_bsa_ 16.5 months.

**Table 2:** semiquantitative PET/CT parameters cutoff calculated using receiver operating characteristic (ROC) curve analysis considering the entire population

	ROC curve for PFS				
Parameter	cutoff	AUC (95% CI)	*p* value	Sens (95% CI)	Spec (95% CI)
PSMA-TV cm^3^	13.3	0.697 (0.620–0.766)	0.004	52.9% (27.8–77)	83% (75.9–88.7)
PSMA-TTV cm^3^	78	0.632 (0.554–0.706)	0.074	52.9% (27.8–77)	70.7% (62.7–78)
PSMA-TL	50	0.841 (0.775–0.893)	< 0.001	64.7% (38.3–85.8)	89.1% (82.9–93.6)
PSMA-TTL	140	0.819 (0.752–0.875)	< 0.001	82.4% (56.6–96.2)	70.1% (62–77.3)
Dmax_bsa_ cm	15.66	0.856 (0.793–0.906)	< 0.001	70.6% (44–89.7)	89.1% (82.9–93.6)

*PFS* progression free survival, *AUC* area under curve, *CI* confidence interval, *sens:* sensibility *spec:* specificity

**Table 3:** univariate and multivariate analyses for PFS

	Univariate analysis		Multivariate analysis	
	*p* value	HR (95% CI)	*p* value	HR (95% CI)
Age	0.180	1.201 (0.900–2.528)		
ECOG performance status	0.445	1.989 (0.728–2.888)		
ISUP	0.949	1.038 (0.382–2.787)		
PSA	0.018	3.459 (1.331–8.989)	0.125	1.528 (0.898–3.669)
LDH	0.568	1.989 (0.700–3.958)		
ALP	0.547	2.582 (0.600–4.564)		
D'amico risk group high	0.663	1.265 (0.438–3.653)		
M + at staging	< 0.001	7.854 (2.828–21.816)	0.021	3.982 (1.229–12.901)
PSMA-TV cm^3^*	0.002	5.729 (1.833–17.907)	0.250	2.546 (0.890–4.001)
PSMA-TTV cm^3^*	0.107	2.281 (0.836–6.227)		
PSMA-TL*	< 0.001	54.519 (14.216–209.085)	0.066	3.102 (0.929–10.374)
PSMA-TTL*	< 0.001	7.269 (2.702–19.532)	0.022	1.580(1.268–1.892)
Dmax_bsa_ cm *	< 0.001	51.148 (14.919–184.340)	0.001	2.078 (1.023–3.136)

*PFS* progression-free survival, *HR* hazard ratio, *CI* confidence interval

*Variables dichotomized using cutoff values after ROC analysis reported in Table 2

PSMa-TV and PSMA-TL; PSMA-TTV and PSMA-TLL were evaluated separately due to the collinearity relationship

## Discussion

The research of early and accurate not-invasive biomarkers to predict treatment response and/or survival are desirable with the aim to maximize treatment efficacy and improve outcome. For PCa patients, PSMA PET/CT had been emerged as a fundamental imaging tool in the staging of high-risk patients, due to the ability to detect distant disease better than conventional imaging including CT and bone scan. Besides visual analysis, several (semi)quantitative parameters derived by PET images are available in the literature. The impact of tumor FDG PET volumetric features on the prognosis of several oncological diseases, especially lymphoma, had been already described [20–22]. These parameters, known as MTV and TLG, reflect at the same time tumor morphological and functional activity of the disease. The same rationale may be applied also in the PCa setting, of course with PSMA-radiopharmaceuticals. First, Schmuck et al. investigated the role of volumetric PSMA variables (PSMA-TV and PSMA-TL) in treatment response evaluation demonstrating a useful yield of these volumetric markers in predicting therapy failure and a linear correlation between these PET features and PSA value [23]. Similar findings were subsequently confirmed by other studies in patients with biochemical recurrence or bone metastases from PCa [24, 25]. But in these cases, only TV and TL were evaluated and not biomarkers expressing global diffuse disease such as PSMA-TTV, PSMA-TTL or Dmax. Instead, PSMA-derived volumetric features calculated at staging PSMA PET/CT were measured and tested only in a few studies [12, 13], showing in all a positive association between PSA and PET features (Table 4). Particularly, PSMA-TTV and PSMA-TTL were demonstrated to be the factors with the highest correlations with PSA. This association is logical because PSA is a protein expressed by cancer cells, thus can be indirectly considered as the expression of tumor burden disease with exceptions in cases of disease not producing PSA. Sometimes also association with age and GG was reported but not confirmed in all papers. This evidence is in agreement with our analysis, where PSMA-TTV and PSMA-TTL were showed to be moderately correlated with PSA and number of the lesions, while no significant correlations with GG were derived.

**Table 4:** the summary of the main studies about the role of semiquantitative PSMA parameters measured at baseline PET/CT

First author	Year	Number of patients	Radiotracer	Main results
Aksu A	2021	88	^68^ Ga-PSMA	PSMA-TTV and PSMA-TTL correlated with PSA. PSMA-TV and PSMA-TL correlated with extension of disease, GG and risk-group
Santos A	2021	46	^68^ Ga-PSMA	PSMA-TTV and PSMA-TTL not correlated with PSA, age, GG
Zschaeck S	2022	135*	^68^ Ga-PSMA	PSMA-TV moderately correlated with PSA and GG
Zou Q	2020	59	^68^ Ga-PSMA	PSMA-TTV and PSMA-TTL correlated with PSA and GG PSMA-TTL independently correlated with PFS
Telli TA	2022	54**	^68^ Ga-PSMA	PSMA-TTV and PSMA-TTL correlated with PSA PSMA-TTV independently correlated with OS

*GG* gleason grade, *PSMA-TV *PSMA prostate tumor volume, *PSMA-TL* PSMA prostate total lesion, *PSMA-TTV* PSMA whole body total volume, *PSMA-TTL *PSMA whole-body total lesion

*not metastatic patients

**mCRPC patients

The prognostic impact of baseline PET volumetric variables in not mCRPC patients in terms of PFS was studied only in one study [13]. In this retrospective work, Zou et al. recruited 59 newly diagnosed PCa and examined the prognostic role of several PSMA PET features (SUVmax, SUVpeak, SUVmean, PSMA-TV, PSMA-TL, PSMA-TTV, PSMA-TTL) showing that only PSMA-TTL was an independent prognostic factor together with GG.

However, volumetric features did not include in their definition the pattern of distribution of disease and the number of lesions PSMA-positive. High-risk PCa patients may have extraprostatic disease and plural distant localizations. On the other hand, Dmax is a feature that symbolizes the distribution and dissemination of disease with increased uptake. The possible advantages of Dmax compared to tumor burden variables are the simpleness, speed of extraction (now automated) and clinical meaning expression of patient-based spatial migration of disease [15]. Furthermore, this parameter is less influenced by technical characteristics such as kind of scanners or acquisition-reconstruction protocols. In this study, we decided to evaluate Dmax normalized by BSA to take into account the size and height of each patient. This normalization was suggested previously by other colleagues in not PCa [20, 21], but it is not yet universally shared. Previous articles [20, 21] that investigated Dmax role in PCa patients considered it as an absolute value without body correction. In the first study [16], Dmax was shown to be strongly correlated with PSA values (rho = 0.793, *p* < 0.001), PSMA-TV (rho = 0.797, *p* < 0.001) and PSMA-TTL (rho = 0.763, *p* < 0.001) and was also influenced by Gleason score grade. Instead, in another research [17] the potential prognostic role of Dmax was tested and compared with PSMA-TV and PSMA-TL demonstrating to be the only independent prognostic factor at multivariate analysis in predicting biochemical recurrence. This finding was concordant with our analysis where we revealed Dmax_bsa_ as a strong predictor of PFS and moderately correlated with PSA value. In our opinion, considering Dmax without normalization for body composition is potentially a limitation because not really representative of disease distribution that is for definition affected by anthropometric characteristics. Particularly, we believe that body composition can affect the effective measurement of Dmax with a potential impact on the extraction of specific thresholds. In our analysis, the combination of PSMA-TTL and Dmax_bsa_ showed to be the better combination to predict survival. Patients with both elevated PSMA-TTL and Dmax_bsa_ demonstrated worse survival than others as shown in Fig. 3. This combination could perhaps be helpful to predict prognosis after first‐line treatment identifying patients with a higher risk of relapse/recurrence. In the clinical scenario, this model might anticipate the imaging follow-up or associate a more aggressive therapeutic plan. Of course, this score needs to be validated in a more solid population, in more events and with a longer follow‐up period.

A difference of our research between previous is the evaluation of [18F]PSMA-1007 than [Ga68]Ga-PSMA PET/CT, the radiotracer used in all studies that previously investigated semiquantitative parameters. [18F]PSMA-1007 and [Ga68]Ga-PSMA had different radiotracer biodistribution: [Ga68]Ga-PSMA PET/CT is characterized by a high urinary clearance that potentially reduces the accuracy in the evaluation of primary/local disease due to the vicinity to radioactive urine. This issue may affect the calculation of TV and TL, which now is automatic but deserves a strong manual check to be sure to avoid to surround not malignant tissues. Of course, the add of a manual modification is for definition operator-dependent and may increase the risk of error. On the other hand, [18F]PSMA-1007 had a hepatic elimination causing a higher easiness to evaluate prostatic disease and pelvic nodes and consequentially to extract TV and TL. However, one of the main limitations of [18F]PSMA-1007 is the risk to have incidental bone uptakes without radiological equivalent, called UBU [5]. To overcome this limit, we applied the BUMP score that showed a good diagnostic performance to distinguish between malignant and non-malignant bone uptake in large populations [4]. However, from a diagnostic point of view different PSMA-tracers do not seem to have statistically different performances in recurrent prostate cancer [26]. However, it is not clear if the calculation of volumetric and dissemination PET features may be significantly affected by the kind of radiotracer.

Larger studies are needed to find the best combination of prognostic variables and the factors most readily used in clinical practice for PCa.

Our research presents several limitations: first, the retrospective nature of the investigation with its known limitations. Second, the relatively short follow-up time due to the recent diffusion in the clinical practice of PSMA PET/CT in our country. Third, the lack of prostatectomy as a procedure in all patients causing the presence of GG derived by biopsy results. Despite this, so far, the present study represents the largest series of PCa patients investigated with volumetric and dissemination parameters derived from staging [18F]PSMA-1007 PET/ CT and their prognostic role.

## Conclusions

In conclusion, in this study we demonstrated that volumetric and dissemination features derived by [18F]PSMA-1007 PET/CT are significantly correlated with clinical features (PSA and number of lesions) and represent useful predictors of PFS.
